# Knowledge of the abortion legislation among South African women: a cross-sectional study

**DOI:** 10.1186/1742-4755-3-7

**Published:** 2006-08-03

**Authors:** Chelsea Morroni, Landon Myer, Kemilembe Tibazarwa

**Affiliations:** 1 Women's Health Research Unit, School of Public Health & Family Medicine, University of Cape Town, Cape Town, South Africa, Faculty of Health Sciences, Anzio Road, Observatory 7925, Cape Town, South Africa; 2 Infectious Diseases Epidemiology Unit, School of Public Health & Family Medicine, University of Cape Town, Cape Town, South Africa; 3 School of Public Health & Family Medicine, University of Cape Town, Cape Town, South Africa

## Abstract

**Background:**

In order to ensure that legalized abortion in South Africa improves reproductive health, women must know that abortion is a legal option in the case of unwanted pregnancy. This study investigated knowledge of abortion legislation eight years after the introduction of legal abortion services in one province of South Africa.

**Methods:**

In 2004/2005, we conducted a cross-sectional study among 831 sexually-active women attending 26 public health clinics in one urban and one rural health region of the Western Cape Province.

**Results:**

Thirty-two percent of women did not know that abortion is currently legal. Among those who knew of legal abortion, few had knowledge of the time restrictions involved.

**Conclusion:**

In South Africa there is an unmet need among women for information on abortion. Strategies should be developed to address this gap so that women are fully informed of their rights to a safe and legal termination of pregnancy.

## Background

Experiences from around the world show that restrictive abortion laws lead women to have unsafe abortions, in turn contributing to over one-tenth of maternal deaths in developing countries [1]. Before liberalization of the South African law in 1996, about 1000 legal abortions were granted annually in South Africa, mostly to middle- and upper-class white women [2]. At the same time, roughly 200,000 unsafe abortions were performed annually, the vast majority among poor black women, resulting in an estimated 45,000 hospital admissions and over 400 deaths from septic abortions each year [2]. The 1996 Choice on Termination of Pregnancy (CTOP) Act gives women in South Africa the right to choose whether or not to have a safe abortion [3]. As a direct result of this legislation, abortion-related morbidity and mortality have plummeted across the country [4]. However, abortion services still remain inaccessible to many women because of stigma, provider resistance, and lack of trained providers and facilities certified by the national or provincial department of health to provide abortions, especially in rural areas [5]; as a result, illegal abortions still occur [6,7].

Abortion is a time-restricted health service: the Act states that a pregnancy may be terminated upon a woman's request during the first 12 weeks of gestation, beyond 12 weeks and up to 20 weeks for reasons of adverse effects on the woman's mental or physical health or socio-ecomomic status, in cases of rape and incest, and in cases where the fetus would suffer from severe physical or mental abnormality. From 20 weeks onwards terminations are available under very limited circumstances. Because of these time restrictions, for this law to fully achieve its goal of improving reproductive health by allowing a woman to decide whether and when to reproduce, women must know that abortion is a legal and accessible option in the case of unwanted pregnancy, ideally *before *they become pregnant. Moreover, women need to be aware of the time constraints involved, as well as how and where to access abortion services. This study investigated knowledge of the abortion legislation eight years after the introduction of legal abortion services in South Africa among women attending primary care public health clinics in the Western Cape Province, South Africa.

## Methods

To assess women's knowledge of key reproductive health services, including abortion, emergency contraception (EC) and voluntary testing and counselling for HIV, in 2004/2005 we undertook a cross-sectional study in 26 community health clinics in one urban and one rural health region in the Western Cape Province, South Africa. The Western Cape is among South Africa's better-resourced provinces, with the largest number of positive reproductive health indicators, and is considered to have the best-developed reproductive health infrastructure in the country. The study was requested by the Western Cape provincial health department. Here we report on women's knowledge about abortion legislation.

In each region, we selected a random sample of primary health care clinics with the probability of selection weighted by patient load based on the clinics' usage statistics obtained from the provincial health department. Over a two-day data collection period at each clinic, interviewers approached consecutive women as they signed into the clinic to participate, regardless of their reason for attending the clinic. Women were eligible if they had ever had sexual intercourse and were between the ages of 15 and 49. The number of women interviewed at each facility was proportional to clinic size (based on total patient load) and varied from 11 to 52. We interviewed consecutive women until the target sample size was reached. Refusals were minimal (<2%). Semi-structured interviews were conducted in participants' home languages and lasted approximately 15 minutes. To assess knowledge of the abortion law, we asked the following key questions: 1) "Does the present law on abortion in South Africa allow for a woman to have an abortion?" 2) "Up to how many weeks of pregnancy is a woman allowed by this law to have a legal abortion?", and 3) "Has a health care worker ever discussed abortion with you?". After responding to these questions, a description of legal abortion was then read to all participants and they were asked open ended questions about their attitudes towards legal abortion and their perceptions of its safety.

In the analysis, responses to open-ended questions were coded and collapsed into categories to facilitate quantitative assessment. Bivariate analyses employed χ-square tests. A multiple logistic regression model was developed to examine how demographic and behavioral factors were associated with abortion knowledge. Variables that demonstrated significant bivariate associations with abortion knowledge (defined as p < 0.05) were entered into the model. Variables were retained in the final model if they demonstrated a significant independent association with the outcome of interest, or if their removal altered the association between other covariates and the outcome of interest [8].

All participants provided written informed consent and ethical approval to conduct the survey was granted by the Provincial Department of Health, the City of Cape Town Health Department, each participating clinic and the Research Ethics Committee of the University of Cape Town.

## Results

Of the 831 women who participated (628 urban participants and 203 rural participants), most were attending the clinic on the day of the interview for medical complaints (37%, n = 307), antenatal or postnatal care (32%, n = 266), or family planning services (21%, n = 175). The median age was 28 years and the median level of education was grade 10 (Table 1). Most participants spoke either Afrikaans or Xhosa as their main language (47%, n = 391 and 44%, n = 366, respectively). Of the 688 participants who had ever been pregnant, 61% (n = 420) reported that their last pregnancy was unintended.

**Table 1:** Socio-demographic characteristics of participants

*Characteristic*	*Urban Region N = 628*	*Rural Region N = 203*	*Total N = 831*
Mean age in years (standard deviation)	28.4 (9.0)	27.3 (8.2)	28.2 (8.9)
Age categories, %			
15–19	18.2	17.9	17.8
20–29	42.6	45.3	43.1
30–39	24.4	28.3	25.2
40–49	14.8	8.5	13.8
Marital status, %			
Married	35.8	31.7	35.4
Single, current relationship	50.8	57.4	53.1
Single, no current relationship	13.3	10.7	11.4
Level of education, %			
No formal schooling	3.1	0.9	2.8
Grade 1-Grade 7 (primary)	15.8	20.8	17.1
Grade 8-Grade 12 (secondary)	77.6	78.3	77.4
Tertiary	3.4	0	2.7
Employment status, %			
Employed	25.5	32.7	27.6
Scholar/student	13.6	9.4	12.3
Unemployed	46.7	44.9	45.7
Houseminder/disabled/pensioner	14.3	13.1	13.8
Main language spoken, %			
Afrikaans	46.0	48.6	46.7
Xhosa	42.7	50.9	44.2
English	10.8	0	8.6
Other	0.5	0.5	0.5

**Table 2:** Assessing associations between participant characteristics and knowledge of legal abortion. A bivariate analysis

*Characteristic, %*	*Know that abortion is legal*	*Do not know that abortion is legal*	*P-value*
Total	68.7	31.3	
Region			
Urban region	71.5	28.5	<0.01
Rural region	60.3	39.7	
Age (years)			
15–19	68.3	31.7	0.5
20–29	68.7	31.3	
30–39	67.0	33.0	
40–49	75.2	24.8	
Education			
Less than secondary school	62.6	37.4	0.1
Secondary school or above	69.4	30.6	
Marital status			
Married	67.0	33.0	0.4
Unmarried	70.0	30.0	
Main language spoken			
IsiXhosa	69.2	30.8	0.6
Afrikaans	67.4	32.6	
English	75.8	24.2	
Other	75.0	25.0	
Last pregnancy unintended			
Yes	69.4	30.6	0.8
No	68.4	31.6	
Method used at last sexual intercourse			
No method	65.0	35.0	<0.01
OC/injectable/sterilization	80.0	20.0	
Male condom/female condom	79.5	20.5	
Heard of EC			
Yes	82.4	17.6	<0.001
No	62.9	37.1	

Overall, thirty-two percent of women (n = 264) did not know that the law in South Africa allows for legal abortion, and this proportion was substantially higher in the rural region (40%, n = 82) compared to the urban region (29%, n = 182) (p < 0.01). Furthermore, from clinic to clinic, the proportion who knew abortion was legal ranged from less than 6% to more than 64%. Among the 567 respondents who were aware of legal abortion, almost half (48%, n = 272) did not know there was a time restriction for a legal termination of pregnancy on request (without restriction). Of the 295 participants who knew that their was a time restriction, 20% (n = 59) thought that it was 12 weeks or less, 4% (n = 12) thought that it was more than 12 weeks, and 76% (n = 224) did not know what the time restriction was. Of those who were aware of legal abortion, only 9% (n = 51) had ever discussed abortion with a health care worker.

Of the total sample, most women perceived legal abortion in the first trimester by manual vacuum aspiration as medically safe (62%, n = 515) and believed that women should be allowed to have a legal abortion upon request (63%, n = 524). A substantial minority of women (38%, n = 316), however, considered legal abortion to be an unsafe procedure, and most commonly mentioned concerns about a reduction in future fertility as the reason.

Age, level of education and employment were not associated with knowledge of legal abortion in the bivariate analysis. In the multivariate analysis, characteristics independently associated with knowledge of legal abortion were: living in the urban vs. rural region (OR 1.5; 95% confidence interval (CI) 1.0–2.0), having heard of emergency contraception vs. having not heard of EC (OR 2.8; 95% CI 1.9–4.2) and having used an effective method of contraception at last sexual intercourse vs. not using contraception (OR 2.0; 95% CI; 1.2–3.3) (Table 3).

**Table 3:** Characteristics independently associated with knowledge of legal abortion (n = 831). A multivaraite analysis.

*Characteristic*	*Odds ratio*	*95% CI*
Region		
Urban region	1.4	1.0–1.9
Rural region	1 (Ref)	
Heard of EC		
Yes	2.8	1.9–4.2
No	1 (Ref)	
Used effective contraception at last sexual intercourse		
Yes	2.0	1.2–3.3
No	1 (Ref)	

## Discussion

This is one of the few studies focusing on South African women's knowledge of the abortion law. These findings show that one-third of women surveyed do not know that abortion is legal in South Africa. Knowledge of the legality of abortion in other similar settings where abortion is legal in some form ranges from 45% in Mexico to 57% in Latvia to 78% in the Gauteng Province of South Africa [7,9,10]. In one qualitative study of South African women who had abortions outside of the legal abortion services, 54% reported having done so because they did not know about the law [7]. The 1998 South African Demographic and Health Survey (DHS), which was conducted less than two years after the implementation of the CTOP Act, found that nationally 53% of women knew of legal abortion; the Western Cape provincial figure was 51% [11]. In this study, 68% of women knew that abortion is a legal health service. Although this study used the same questions as the DHS, the DHS figures are not directly comparable to these findings due to different sampling methodologies: the DHS was a community-based sample of women and this study sampled women attending health services. A comparison of these two sets of data suggests that more women know about legal abortion now than did in 1998. However, another explanation for this apparent difference in levels of knowledge is that this survey was conducted among individuals attending public health clinics, with greater access to health education. Thus, the 2004/2005 results may simply reflect greater knowledge in this sample compared to the general population of the Western Cape, as opposed to an increase in knowledge among all women through time. In general, it is likely that awareness of abortion legislation in this clinic-based sample in the Western Cape Province, which has a better reproductive health infrastructure than most other areas of the country, is higher than in the general population of South Africa.

These data suggest that approximately one-third of the women we surveyed in 2004/2005 do not know that abortion is legal in South Africa. This finding, coupled with the findings that 61% of last pregnancies in this sample were unintended and 25% of women who did not want to fall pregnant did not use contraception during last intercourse [12], is worrisome. Not only are an appreciable proportion of these women uninformed about the option of abortion in the case of unwanted pregnancy, they are also unable to protect themselves from unintended pregnancy in the first place. This study shows that lack of knowledge of legal abortion is associated with lack of other reproductive health knowledge, such as awareness of EC and contraceptive use. Thus, the 32% of women who do not know that abortion is a legal option may be the women at greatest risk for unwanted pregnancy.

Furthermore, this study reveals tremendous variability from clinic to clinic in terms of women's knowledge of the abortion legislation. Reasons for this inter-clinic variability are poorly understood. This is a key finding that requires further research so that the health services are able to appropriately target certain clinics and areas for intervention.

Given that only 9% of those aware of the law had ever discussed abortion with a healthcare worker, there is clearly a need for greater client-provider dialogue regarding abortion, particularly the time restrictions and safety of the legal procedure. Regardless of individual provider beliefs, relaying basic information on the legality of abortion may need to become part of routine reproductive health counseling.

In addition, community-based health information campaigns and school-based sex education and life skills programs should incorporate information on abortion services. Expanding access to information about abortion beyond the clinic setting is essential in that women who are at highest risk for unintended pregnancy and therefore for abortion – women who cannot or do not access family planning services – are unlikely to visit a healthcare provider who could discuss the law with them.

## Conclusion

Overall, these findings indicate that there is a substantial unmet need among women for information on abortion. Strategies should be developed to address this gap so that women are fully informed of their rights to a safe and legal termination of pregnancy. For the abortion legislation to fully contribute to improve health in South Africa, all South African women must know that abortion is a legal and accessible option in the case of unwanted pregnancy.

## Competing interests

The author(s) declare that they have no competing interests.

## Authors' contributions

CM conceptualised and designed the study, oversaw data collection and data analysis, was primarily responsible for the interpretation of results and drafting the manuscript.

LM participated in designing the study, conducted the data analysis and participated in the interpretation of results and drafting and critically reviewing the manuscript.

KT assisted in data analysis, interpretation of results and critically reviwed the manuscript.

All authors read and approved the final manuscript.
